# The Mediating Role of Visual Stimuli From Media Use at Bedtime on Psychological Distress and Fatigue in College Students: Cross-Sectional Study

**DOI:** 10.2196/11609

**Published:** 2020-03-16

**Authors:** Yuan Guan, Wenjie Duan

**Affiliations:** 1 Department of Social Work Guangdong University of Finance Guangzhou China; 2 Social and Public Administration School East China University of Science and Technology Shanghai China

**Keywords:** psychological distress, visual stimulus, auditory stimulus, bedtime media use, fatigue

## Abstract

**Background:**

Empirical research has linked psychological distress with fatigue. However, few studies have analyzed the factors (eg, stimuli from bedtime media use) that affect the relationship between psychological distress and fatigue.

**Objective:**

The aim of this study was to examine whether visual stimuli from bedtime media use mediate the relationship between psychological distress and fatigue among college students.

**Methods:**

The sample included 394 participants (92 males, 302 females) with a mean age of 19.98 years (SD 1.43 years), all of whom were Chinese college students at an occupational university in Sichuan Province, China. Data were collected using a paper-based questionnaire that addressed psychological distress, stimuli from bedtime media use, and fatigue. Mediation analysis was conducted using the PROCESS macro version 2.16.2 for SPSS 22, which provided the 95% CIs.

**Results:**

Both psychological distress (r=.43, *P*<.001) and visual stimuli from bedtime media use (r=.16, *P*<.001) were positively related to fatigue. The association between auditory stimuli from bedtime media use and fatigue was not significant (r=.09, *P*=.08). The relationship between psychological distress and fatigue was partially mediated by visual stimuli from bedtime media use (beta=.01, SE 0.01, 95% CI 0.0023-0.0253).

**Conclusions:**

The findings imply that psychological distress has an indirect effect on fatigue via visual stimuli from bedtime media use. In contrast, auditory stimuli from bedtime media use did not have the same effect. We suggest that college students should reduce bedtime media use, and this could be achieved as part of an overall strategy to improve health. Mobile health apps could be an option to improving young students’ health in daily life.

## Introduction

### Fatigue and Its Influence

Fatigue is a subjective feeling of tiredness or a sustained sense of exhaustion [1,2]. It includes the experience of fatigue and the influence of fatigue on physical, mental, and social aspects of life [1,2]. Fatigue is common among students in China [3]. In a study of 757 Chinese adolescent students in Taiwan, Chen et al [4] found relatively high proportions of fatigue in grades 9 through 12, primarily owing to the pressure students faced related to university entrance exams. Fatigue has a negative influence on students’ school performance [5] and health [6]. For example, using data from 60 college-aged students, Palmer [7] found that fatigue had a negative influence on students’ learning and cognitive performance. In their study of 109 medical students aged 21-40 years, Hwang et al [8] found that students with clinical fatigue had low scores for physical health and psychological health.

### Fatigue and Related Research

Fatigue has attracted considerable research interest in recent years. The large proportions of students who experience fatigue and the serious consequences of fatigue highlight the need to explore the psychological mechanisms that underlie fatigue. Although most of the published studies have attempted to estimate the negative consequence of fatigue, relatively few studies have analyzed the factors influencing fatigue. Some research has focused on the risk factors for fatigue, such as daily activities [9] and daily events [10]. However, only a few studies have investigated the factors influencing fatigue, such as bedtime media use. Using data from 358 university students, Zarghami et al [11] found that using a cell phone after switching off the main bedroom light was associated with fatigue. Also, relatively few studies have explored the relationship between psychological distress, an important influencing factor and intervening variable, and fatigue among college students in China.

### Psychological Distress and Fatigue

Psychological distress is a composite concept that describes the negative symptoms of a person’s mental health, such as depression, anxiety, or other emotional dysregulation [12,13]. Psychological distress is positively related to fatigue [4,14,15]. Based on one-way ANOVA analysis of data from a cross-sectional survey of 355 first-year rural college students, Hussain et al [16] found that fatigued students experienced higher levels of psychological distress. In a longitudinal study of 243 college students in South Korea, Shim et al [17] found that depression is positively associated with fatigue.

### Media Use and Psychological Distress

Despite the association between psychological distress and fatigue, the mediating mechanism related to bedtime media use has not been explored. Research on media use has recently become a hot topic. Media use is prevalent among youth [18,19], especially at bedtime [20]. Psychological distress is related to media use [21-23] and positively influences media use [24,25]. For example, in their study with 923 college students in Jiangxi Province, China, Ye and Zheng [26] found that psychological distress resulted in higher media use. Wills et al [27] found that addictive forms of behavior were related with an inability of adolescents to control their emotions. Failure to control negative emotions leads to increased media use [28]. According to the social cognitive theory, self-regulation is a process in which people control their behaviors [29]. Failure to affective self-react (one of the processes of self-regulation) is associated with negative outcomes, such as addiction to media use [30]. Therefore, people who experience psychological distress tend to use media to comfort themselves and, especially at bedtime, to relieve psychological distress [31]. Meanwhile, according to the uses and gratifications theory [32,33], people achieve gratification when they meet their needs, and using specific types of media provided this gratification [33]. Psychological differences are one factor that drives individuals to gratify their needs [24,33]. Therefore, psychological distress is positively associated with media use [34].

### Media Use and Fatigue

Many studies have shown that media use is related to fatigue [20,35-39], and frequent media use results in fatigue [40]. Bedtime media use leads to a later time to fall asleep and displaced sleeping times, which result in fatigue [20]. Moreover, bedtime media use results in irregular sleep [41], disrupting the endocrine system [42]. Students who use media at bedtime may experience two types of stimuli: visual and auditory. Bedtime media use increases the level of external stimuli (visual stimulus and auditory stimulus) at bedtime. Exposure to light at night influences the human biological clock [43,44], and visual stimuli at bedtime influence the neuroendocrine system [45], affecting sleep and resulting in fatigue the following day. Although stimulation at bedtime influences the neuroendocrine system and therefore fatigue [42], few studies have found an association between an auditory stimulus from bedtime media use and fatigue. However, there is limited evidence for an association between listening to music at bedtime and fatigue. With a sample of 844 adults (18-94 years old), Exelmans and Van den Bulck [20] found that listening to music before sleeping increases fatigue. The relationship between an auditory stimulus from bedtime media use and fatigue needs further research.

### Hypotheses

**Figure 1:**
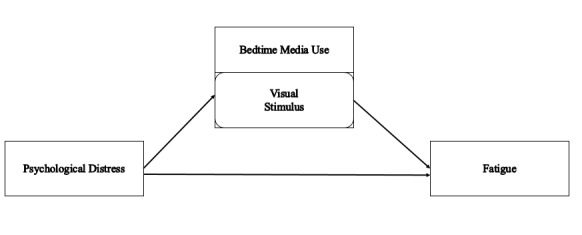
Theory model of the mediating effect of a visual stimulus from bedtime media on the relationship between psychological distress and fatigue.

In summary, psychological distress has a positive relationship with fatigue. Visual stimuli from bedtime media use have a potential mediating role between psychological distress and fatigue. This study aimed to explore the mediating effect of stimuli from bedtime media use on the relationship between psychological distress and fatigue among Chinese college students. First, we aimed to confirm the results of previous studies by testing the relationship between psychological distress and fatigue. Second, we aimed to extend the existing literature by examining the role of bedtime media use in the relationship between psychological distress and fatigue. We had two hypotheses: (1) psychological distress is positively correlated with fatigue, and (2) visual stimuli from bedtime media use mediate the positive correlation between psychological distress and fatigue. This paper not only provides new insight in this area but also provides practical methods to improve college students’ health in China. The theory model is shown in Figure 1.

## Methods

### Participants and Procedures

**Figure 2:**
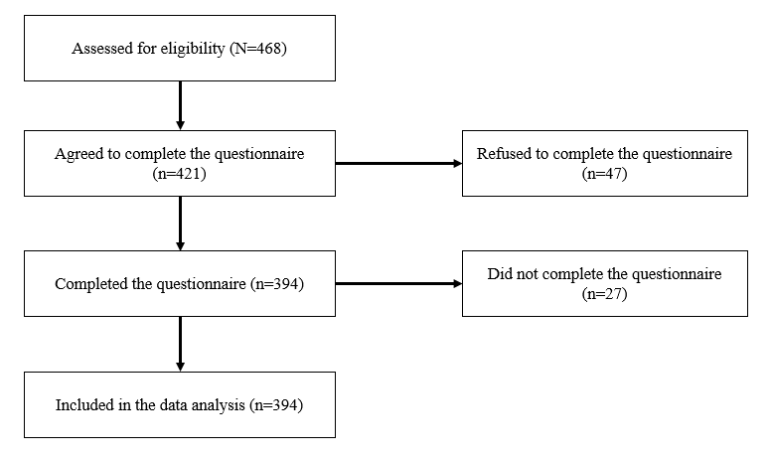
Flow chart of eligibility and participation.

A power analysis was conducted to estimate the minimum sample size using G Power software [46]. The regression model included 5 predictors (sex, age, psychological distress, visual stimulus, and auditory stimulus), the alpha was set at .05, and the observed R^2^ in the regression model was 0.21 (Table 1). The minimum sample size was calculated at 158 participants. The power was 0.95, which was an adequate value [47]. The participants were 468 freshmen from an occupational university in Sichuan Province, China. The inclusion criteria were age between 18 and 30 years old and owning a media device for daily use. All participants were asked to complete a questionnaire; 10.0% (47/468) of the participants refused to complete the questionnaire, and 6.4% (27/421) of the remaining participants did not complete the questionnaire. The data were collected in 2016, and 394 completed questionnaires were collected. All data were collected in the form of paper-based questionnaires. The flow chart of eligibility and participation is shown in Figure 2. A normal Q-Q plot and histogram showed that the fatigue scores were approximately normally distributed. The necessary ethical approval was obtained from the Human Research Ethics Committee of the Department of Sociology, Wuhan University, China, and the research was conducted in accordance with the Declaration of Helsinki. Written informed consent was obtained from all the participants.

**Table 1:** Results from the hierarchical regression analyses of the effects of the demographic variables, psychological distress, and stimuli from bedtime media use on the dependent variable, fatigue.

	Step 1			Step 2			Step 3		
	beta	*t*	*P* value	beta	*t*	*P* value	beta	*t*	*P* value
Constant	47.15	12.68	<.001	46.70	13.80	<.001	45.29	13.14	<.001
Sex (male, female)	–1.61	–2.57	.01	–1.14	–1.99	.047	–1.46	–2.50	.01
Age (years)	0.29	1.56	.12	0.12	0.69	.49	0.09	0.54	.59
Psychological distress				0.25	9.04	<.001	0.24	8.65	<.001
Visual stimuli							0.92	2.59	.01
Auditory stimuli							–0.18	–0.64	.52
*R* ^2^	.02			.19			.21		
*F* statistic	*F*_(2,391)_=4.34			*F*_(3,390)_=30.76			*F*_(5,388)_=20.27		
*P* value	.01			<.001			<.001		

### Measures

#### Psychological Distress

The Depression Anxiety Stress Scales (DASS) can be used to measure general psychological distress [48]. As a self-reported instrument, the DASS measures the three related psychological states of depression, anxiety, and stress [49]. It is a 4-point scale that asks participants about their experiences in the past week (eg, “I felt that life was meaningless”), with scores ranging from 0 (“This item does not apply to me at all.”) to 3 (“This item applies to me very much.”). No item is reverse-coded. The short version, DASS-21, has half the questions of the DASS-42 and is suitable for research use rather than clinical use [50]. The total score of the DASS-21 provides a composite/general measure of the negative psychological distress of the general population rather than for people with a specific psychological state [50]. Therefore, the DASS-21 was suitable for this exploratory study, which did not measure the endocrine index as is done in a biomedical experiment. Research has shown that the psychometric properties of the Chinese version of the DASS-21 is acceptable [51]. The Cronbach alpha of the current sample was .89. The total score of the DASS-21 was used to measure psychological distress among college students.

#### Bedtime Media Use

Based on previous studies [20,38,39,52,53], we asked the respondents how often they used media to help them fall asleep (eg, surfed the internet or watched videos) and about the two types of stimuli (ie, auditory stimulus and visual stimulus) before bedtime. Visual stimuli included playing computer games, watching videos, or surfing the internet on a computer; playing computer games, watching videos, or sending short messages on a smartphone; and reading. Auditory stimuli included listening to music and making phone calls. For example, we asked how often the respondent played computer games to help them fall asleep, with responses on a 6-point scale, from 1=never to 6=always. For the statistical analyses, the mean scores of the 7 visual stimulation activities and the mean scores of the 2 auditory stimulation activities were calculated.

#### Fatigue

The PROMIS Fatigue Short-Form 7a measures self-reported fatigue using a single total score [1]. The 7 questions (eg, “How often did you experience extreme exhaustion in the past seven days?”) each have a possible score from 1 (never) to 5 (always). The Fatigue Short-Form 7a scale is available to use with college students (age ≥18 years), and the Chinese version is available from PROMIS [54]. The psychometric properties of the PROMIS scale have been tested [55]. The Cronbach alpha of fatigue was .78. For the statistical analyses, the total score of the 7 items was transformed to a standardized T score (mean 50 points, SD 10 points) using the coding from PROMIS [54]. As a self-reported instrument, the PROMIS Fatigue Short-Form 7a is available to assess fatigue for people without a clinical condition such as general-population university students.

### Data Analysis

The pairwise method was used to handle missing data. First, descriptive statistics and the correlation matrix were calculated. We expected that psychological distress, stimuli from bedtime media use, and fatigue would be positively related to each other. Second, hierarchical regressions were performed using the entry method to explore the roles of psychological distress and stimuli from bedtime media use on fatigue. In the hierarchical regression, fatigue acted as the dependent variable. Demographic variables (ie, age and sex, coded as 1 for male and 0 for female) were entered in step 1, followed by psychological distress in step 2. The two types of stimuli from bedtime media use were entered in step 3. Third, using the results from the hierarchical regressions, the mediating effect was examined using the PROCESS macro version 2.16.2 for SPSS 20.0 (IBM Corp, Armonk, NY) [56]. Bootstrapping was set at 5000 resamples to provide robust estimates of the 95% CIs of the standardized effects [57]. We examined if the stimuli from bedtime media use mediated the association between psychological distress and fatigue. A model was constructed with psychological distress as the predictor (X), fatigue as the outcome (Y), and visual stimulus from bedtime media use as the mediator (M). Covariates included sex and age. Our model describes path a as the direct effect of the psychological distress regressed on the visual stimuli from bedtime media use. Path b is the direct effect of visual stimuli from bedtime media use regressed on fatigue. Path c is the direct effect of psychological distress regressed on fatigue. Path c’ is the mediating effect of psychological distress regressed on fatigue through visual stimuli from bedtime media use (c’ = a * b). *P* values are two-tailed, and the statistical significance level was set at *P*<.05.

## Results

### Sample Characteristics

The mean age was 19.98 years (SD 1.43 years, range 18-26 years), and 76.6% (302/394) of the participants were female.

### Bivariate Correlation Analyses

The mean (SD) points for visual stimuli from bedtime media use, the auditory stimuli from bedtime media use, psychological distress, and fatigue were 3.08 points (0.84 points), 3.76 points (1.04 points), 15.19 points (8.89 points), and 52.55 points (5.32 points), respectively. Visual and auditory stimuli from bedtime media use had a significant positive relationship (r=.56, *P*<.001). Psychological distress had a significant positive relationship with fatigue (r=.43, *P*<.001). Psychological distress also had a significant positive relationship with visual stimuli from bedtime media use (r=.13, *P*=.009), while the relationship between psychological distress and auditory stimuli from bedtime media use was not significant (r=.10, *P*=.059). Visual stimuli from bedtime media use had a significant positive relationship with fatigue (r=.16, *P***<**.001), while the relationship between auditory stimuli from bedtime media use and fatigue was not significant (r=.09, *P***=**.08). Therefore, the effect of visual and auditory stimuli from bedtime media use on psychological distress might differ. And, the two types of stimuli from bedtime media use might have different effects on fatigue.

### Hierarchical Regression Analyses

The results of the hierarchical regression analyses are shown in Table 1. All regression equations were statistically significant (*F*_(2,391)_>4.34, *P*<.05). Psychological distress was positively related to fatigue (t_390_=9.04, *P*<.001). The two types of stimuli from bedtime media use were entered in step 3, and the results showed that visual stimuli had a small yet significant explained variance on fatigue (t_388_=2.59, *P*=.01). The effect of auditory stimuli regressed on fatigue was not significant (t_388_=–.64, *P*=.52). These results support hypothesis 1, that psychological distress and visual stimuli from bedtime media use positively affected fatigue.

### Analyses of Mediating Factors

**Figure 3:**
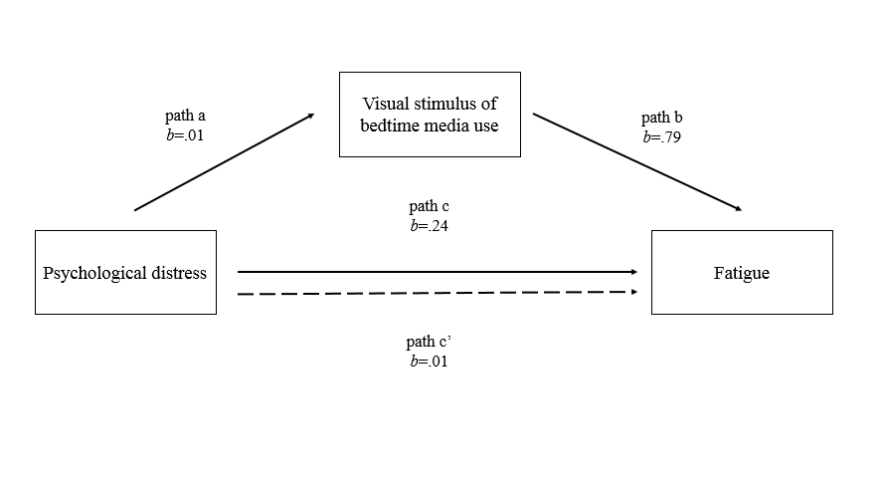
Direct effects (paths a, b, and c) of psychological distress and visual stimuli from bedtime media use on fatigue and an indirect effect (path c’) of psychological distress on fatigue through the visual stimuli from bedtime media use. The control variables were age and sex.

The results of the analyses of the mediating effects of stimuli from bedtime media use are shown in Table 2. As shown in Figure 3, psychological distress was significantly related to visual stimuli from bedtime media use (path a: beta=.01, SE 0.005, *P*=.006). Bedtime media use was significantly correlated with fatigue (path b: beta=.79, SE 0.29, *P*=.007).

Based on the bivariate analysis, psychological distress was correlated with fatigue (path c: beta=.24, SE 0.03, *P*<.001) when controlling for visual stimuli from bedtime media use. Consistent with hypothesis 3, there was an indirect effect of psychological distress on fatigue through the visual stimuli from bedtime media use (path c’: beta=.01, bootstrapped SE 0.01, bootstrapped 95% CI 0.0023-0.0253). Therefore, the positive association between psychological distress and fatigue was partially mediated by visual stimuli from bedtime media use.

**Table 2:** Mediating effect of the visual stimuli from bedtime media use on the relationship between psychological distress and fatigue.

Path	*R* ^2^	beta	SE	*P* value	95% CI
Psychological distress and visual stimuli	.05	.01	0.01	.006	0.0039-0.0225
Visual stimuli and fatigue	.21	.79	0.29	.007	0.2152-1.3622
Psychological distress and fatigue (direct effect)		.24	0.03	<.001	0.1842-0.2926
Psychological distress and visual stimuli and fatigue (indirect effect)		.01	0.01		0.0023-0.0253

## Discussion

### Study Objective

To improve college students’ health and ability to study efficiently, fatigue should not be neglected, especially when fatigue is associated with psychological health. This study explored the association between psychological distress and fatigue. The mediating mechanism of bedtime media use was also analyzed.

### Discussion of Findings Regarding the Hypotheses

In line with our first hypothesis, there was a significant positive association between psychological distress and fatigue, which is consistent with results from previous studies [58-60]. It is conceivable that increasing psychological wellbeing and maintaining a good mood positively affect physiological systems and improve college students’ vitality. Psychological interventions may help students maintain mental and physical health and increase their learning efficiency [61].

Supporting our second hypothesis, the association between psychological distress and fatigue was mediated by visual stimuli from bedtime media use. Due to the pressure from learning activities, it is common for young students to have mental health concerns [62]. Media use is a common method to regulate emotion [63]. Students with psychological distress increase their media use as a method for emotional regulation. This makes them feel comfortable and gratified [64]. However, visual stimuli from bedtime media use influence the endocrine system, disrupt the biological clock, and replace sleeping time. This potentially influences fatigue during the day. Students’ schedules are restricted by school hours. Students who fall asleep later at night because of media use at bedtime may not compensate by getting up later and therefore not get enough sleep. This results in fatigue the next day. In this study, the relationship between auditory stimuli from bedtime media use and fatigue was not significant. Therefore, the effect of an auditory stimulus from bedtime media use might not be enough to disrupt sleep and cause fatigue among college students.

This paper provides practical strategies to improve the health of college students. First, administrators should consider the negative effect of stimuli from bedtime media use and provide students with an intervention to control bedtime media use. Equally important is students’ awareness of ways to promote mental health. Educators who work to improve students’ emotion regulatory abilities could teach skills to relieve psychological distress and address habitual excessive bedtime media use, such as through the practice of mindfulness [65]. This would help students improve their sleep quality [66] and decrease fatigue. Second, to reduce fatigue and improve study efficiency among college students, reducing bedtime media use, particularly the use of a smartphone screen, could be a good strategy to relieve psychological distress. Mobile health apps could be a good option to improve college students’ health and working efficiency. Some mindfulness training apps, such as Headspace, provide courses to improve health through auditory resources only. App-based mindfulness training positively affects emotional regulation and sleep [67]. It could be a suitable option for college students to improve their health and avoid additional visual stimuli at bedtime.

### Limitations and Implications for Future Research

Several limitations of this study should be noted. First, the sex distribution and generalizability need to be considered. Further data are needed to extend the results to students outside of China. Future research should improve the representativeness of the sample. Second, the items to measure media use and the content of media use need improvement, and further investigation is required. Although there were 7 items to measure visual stimuli from bedtime media use, there were only 2 items to measure auditory stimuli from bedtime media use. This might affect the validity of the results. As a result, future studies should improve the sample and measurements. Third, parents are an important influence on college students’ daily behavior and mental health. Future research should consider controlling the effects of parental education level and occupation.

### Conclusions

This paper contributes and expands the current research about the effects of stimuli from bedtime media use and psychological distress on fatigue among Chinese college students. Specifically, the results suggest that visual stimuli from bedtime media use can serve as a mediating factor to understand the association between psychological distress and fatigue. Further research should investigate the effects of the type (ie, playing games and social communication) of bedtime media use on mental and physical health.

## Abbreviations

DASS: Depression Anxiety Stress Scale
